# Use of mobile health units in natural disasters: a scoping review

**DOI:** 10.1186/s12913-024-12067-9

**Published:** 2025-03-12

**Authors:** Sarika Sheerazi, Sarah Ahmad Awad, Johan von Schreeb

**Affiliations:** Department of Global Public Health, Karolinska Institutet, Solnavägen, 17177 Stockholm, Sweden

**Keywords:** Mobile Health Unit, Emergency Medical Team, Natural Disaster, Disaster Response

## Abstract

**Background:**

Mobile Health Units have become important resources for healthcare delivery to dispersed populations following natural disasters. However, criticism regarding their operational flexibility, health coverage and cost-effectiveness remain unaddressed. There are few studies evaluating their usefulness in natural disasters and deployment reports have never been included in peer-reviewed publications. With an expected rise in weather-related disasters, knowledge about the impact of MHUs on addressing health needs is needed. This study aimed to elucidate the use of mobile health units in natural disasters as described in the literature.

**Methods:**

A scoping review was conducted, searching twenty-six databases and websites. Documents detailing operational characteristics and practices of mobile health units deployed to natural disasters, published between 2000 and 2022 in English, were included. Findings were analysed using thematic content analysis with the World Health Organization Classification and Minimum Standards for Emergency Medical Teams as a guiding framework.

**Results:**

Nearly 3000 documents were screened, yielding thirteen documents eligible for inclusion. The literature highlighted seven themes: key characteristics, operational availability, services, benchmark indicator, staff, self-sufficiency and pre-deployment preparations. The reports cover earthquakes, floods, tsunamis, hurricanes, typhoons, cyclones, landslides and mudslides. Mobile health units were described to improve access to outpatient healthcare for populations with limited access to routine services. However, limitations related to mobility, logistics, referral capacity, health coverage and communication posed significant challenges.

**Conclusions:**

Data on the use of mobile health units in natural disasters is scarce with inconsistent reporting of key aspects, stressing a need for uniform reporting. In response to inaccessible fixed healthcare facilities, mobile health units were described to address the normal burden of disease rather than emergency care. Coordination, transportation, referral systems and data collection were highlighted as the main areas of improvement.

**Trial registration:**

Not applicable.

## Introduction

Disasters have profound impacts, overwhelming communities and stretching healthcare systems beyond their limits [1, 2]. This is particularly pronounced in low- and middle-income countries and areas with vulnerable infrastructure and irregular urban settlements [3–5]. The frequency and severity of weather-related disasters have increased over the past two decades [1]. While the immediate effects of natural disasters may be trauma related, the main health impacts are attributed to non-communicable-diseases (NCDs), communicable-diseases (CDs), mental health conditions and maternal and child health (MCH) due to disruption of regular healthcare services [6–9]. However, damage to healthcare facilities and disruption of medical supply chains may leave a vacuum of care in the aftermath of such events, compelling the need for assistance [10, 11].

International healthcare assistance may be deployed to disasters [10]. However, it has been criticized for arriving too late, being uncoordinated and over-focused on trauma care [4, 6, 12–14]. To improve quality and coordination of healthcare assistance in disasters, the World Health Organization (WHO) launched the Emergency Medical Team (EMT) Initiative in 2013 [10]. The initiative provides minimum standards (Blue book) for EMTs in disasters [10]. Emergency medical teams are categorized into four types (I-IV), wherein type I is available in both fixed (EMT type 1 fixed) and mobile (EMT type 1 mobile) capacities [10]. Type 1 mobile EMTs are classified mobile health units (MHUs) that provide sector coverage in hard-to-reach areas rather than single site deployments [10]. Characterized by the ability to provide healthcare services across multiple locations, the mobile mode of health service delivery is highlighted as a flexible option for healthcare provision to displaced populations [7, 10, 15].

The WHO describes them as a “good illustration of the tension between equity of access and the efficient utilization of scarce human resources” [16]. However, concerns about their impact on addressing health needs in natural disasters have emerged [7, 17, 18]. Previous deployments report challenges related to transportation, coordination, health coverage, cost-effectiveness and funding [7, 15, 17]. The ICRC have described the mobile health service modality as “expensive to run” and a “logistical nightmare” [15]. The systematic review on mobile clinics in humanitarian emergencies by McGowan et al. (2020) supported these concerns and further highlighted a lack of data in peer-reviewed literature [7]. However, with the rigid eligibility criteria of systematic reviews, only five studies were included in this review, compelling the need for a broad and more inclusive approach for gathering information from disaster contexts [19].

The Sendai Framework on Disaster Risk Reduction 2015–2030 highlights the importance of continuous evaluations of healthcare interventions in disasters to optimally tailor them to health needs [20]. Standardized data reporting protocols, such as the Emergency Medical Team Minimum Data Set (EMT MDS) and adaptations of the Utstein-style template, have been developed in efforts to improve data collection in disasters [21, 22]. However, deployment reports and operational summaries have never been included in peer-reviewed publications addressing the role of MHUs in natural disasters [7]. With a projected rise in weather-related disasters, the impact of MHUs on filling healthcare gaps in natural disasters remains poorly understood [7]. To guide the EMT Initiative and improve the capacity of MHUs to address healthcare needs in natural disasters, more knowledge is needed [1, 7, 10].

## Aims

This study aimed to elucidate the use of mobile health units in natural disasters as described in the literature.

## Materials and methods

### Study design

Due to the limited data in peer-reviewed publications, this study was conducted following the scoping review methodology of Arksey and O’Malley updated by the Joanna Briggs Institute (JBI) for scoping reviews [23, 24]. Scoping reviews are used to systematically map literature and identify knowledge gaps in broader topics rather than providing specific answers to narrow research questions [25]. By allowing inclusion of grey literature, such as operational reports and expert evaluations, the scoping review approach facilitates comprehensive mapping of literature from disaster settings [24].

### Search terms

A search term list was produced by collection of MeSH terms and key words for mobile health units and natural disasters following an initial search on PubMed, Web of Science, EM-DAT, OpenGrey and Global Heath Observatory in September 2021. The search terms are displayed in Appendix A. The most common types of natural disasters from the past four decades, reported by the Centre for Research on the Epidemiology of Disasters (CRED) and the United Nations Office for Disaster Risk Reduction (UNDRR), were included [1]. Search term Group A consisted of search terms for natural disasters and Group B consisted of search terms for mobile health units. The compilation of search terms was assisted by librarians at Karolinska Institutet.

### Search strategy

The bibliographic search engines Campbell Collaboration, CINAHL, Cochrane Library, Embase, Global Health, Medline, PubMed and Web of Science were queried using both search term groups. The filters “All text” and “Multi-field search” were applied on the bibliographic databases since the title and abstract filters rendered few hits. Search strategies for databases of organizations involved in disaster response were adopted according to database format due to the lack of advanced settings. Each search term in Group A was queried individually in the database of the African Religious Health Assets Programme (ARHAP), Global Health Observatory (GHO), International Committee of the Red Cross (ICRC), Médecins Sans Frontières (MFS) Analysis, MSF Centre de Réflexion sur l'Action et les Savoirs Humanitaires (CRASH), MSF Research Unit on Humanitarian Stakes and Practices (UREPH) and Relief Web.

Terms for MHUs were excluded from searches on most grey literature databases due to the inability to combine multiple search fields. Since this adaptation was applied on databases of organizations already working with healthcare provision in natural disasters, it was assessed that the adaptation would not compromise the final literature extraction. Search strategy adaptations were discussed between the authors until consensus was reached.

The search strategy was costumed for Google Scholar due to the character limit and inability to apply truncation. Search term Group B was divided into smaller groups for each disaster type, yielding eleven groups. The first 300 results of each search were reviewed using the titles and short texts below. The number of screened hits was chosen to capture the most relevant results while still being a feasible number to review.

### Eligibility assessment and data analysis

Eligibility criteria included documents describing operational characteristics of mobile health units in natural disasters published between 2000 and 2022 in English. The eligibility criteria are displayed in Table 1. Excluded documents mainly described clinics that were reported to be mobile but only operated in one site throughout the deployment. Documents meeting the eligibility criteria were included for thematic content analysis (TCA). The analysis consisted of an initial categorization of data into information units with Microsoft Excel Spreadsheet Software. Related information units were grouped into codes. Codes addressing similar aspects of MHU operations were grouped into themes. The information focus of each theme was labelled according to WHO Classification and Minimum Standards for Emergency Medical Teams to ascertain established terminology. If corresponding WHO indicators were not identified, the theme was titled with a suitable name reflecting its content after discussion between the authors.

**Table 1:** Eligibility criteria

	**Inclusion**	**Exclusion**
Article type	Operational reports, deployment reviews, peer-reviewed studies	News articles, blog posts, documents with limited traceability
Year of publication	2000—2022	
Language	Full text in English	
Setting	Earthquake, flood, cyclone, hurricane, typhoon, tornadoes, earthquake, tsunami, landslide, rockslide, mudslide, drought, wildfire, volcanic eruption, avalanche, dry mass movement, extreme temperature, extreme winter condition, rockfall	Settings otherwise unrelated to natural disasters, such as conflict settings
Population	Directly affected by natural disasters	
Operational characteristics	MHU visited more than one location during the deployment	Specialized MHUs

## Results

**Fig. 1:**
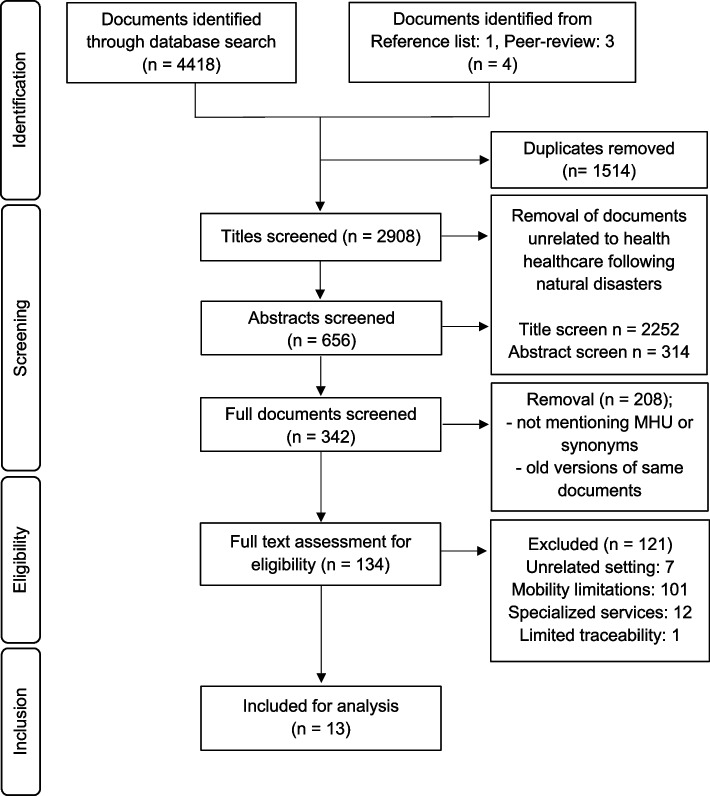
PRISMA Flow Chart

The search yielded 4422 documents for screening, resulting in 10 peer-reviewed publications and 3 deployment reports eligible for inclusion. An overview of the search strategy is displayed in Fig. 1 PRISMA Flow Chart.

### Source characteristics

Mobile health units were the primary objects in 7 documents while 6 documents described an overall response with different modalities of healthcare delivery. The documents were produced by teams operating in earthquakes, floods, tsunamis, hurricanes, typhoons, cyclones, landslides and mudslides. All 3 deployment reports were published by the International Federation of Red Cross and Red Crescent Societies (IFRC). An overview of the included documents is presented in Table 2.

**Table 2:** Source characteristics

Lead author and Year	Classification	Method	Setting	Target population	Aim
Ahmad 2008 [26]	Peer-reviewed	Operational review	Flood in Johore, Malaysia 2006	All affected	Describe experiences of a MHU
Bouland 2019 [27]	Peer-reviewed	Operational review	Hurricane (Dorian) in Abaco, Bahamas 2019	All affected	Describe experiences of a classified EMT Type 1 Mobile (Team Rubicon)
Broach 2010 [28]	Peer-reviewed	Operational review	Earthquake in Port-au-Prince, Haiti 2010	Displaced individuals in tent camps	Describe injury-illness profiles of patients treated in a MHU
Ho 2016 [29]	Peer-reviewed	Operational review	Earthquake in Kathmandu, Nepal 2015	All affected	Describe experiences of SAF
IFRC Bangladesh 2014 [30]	Grey literature	Deployment report	Flash floods and landslides in Bangladesh 2012	All affected	Summarize relief efforts of a collaborative response by IFRC and BDRCS
IFRC Colombia 2017 [31]	Grey literature	Deployment report	Landslide and mudslide in Mocoa, Colombia 2017	All affected	Summarize relief efforts of a collaborative response by ICRC, IFRC, UNGRD
IFRC Pakistan 2017 [32]	Grey literature	Deployment report	Flood in Baluchistan, Pakistan 2017	All affected	Summarize relief efforts of a collaborative response by IFRC, PRCS, NDMA, PDMA
Kim 2010 [33]	Peer-reviewed	Operational review	Cyclone (Nargis) in Myanmar 2008	All affected	Assess the epidemiology of patients observed by a Korean DRT
Krol 2007 [34]	Peer-reviewed	Operational review	Hurricane (Katrina) in Mississippi, USA 2005	Underserved populations*	Describe experiences of a mobile medical care approach
Lateef 2009 [35]	Peer-reviewed	Operational review	Cyclone (Nargis) in Yangoon, Myanmar 2008	All affected	Describe experiences of a MHU
Li 2012 [36]	Peer-reviewed	Operational review	Tsunami in Aceh, Indonesia 2004; Earthquake in Yogyakarta, Indonesia 2006; Earthquake in Port-au-Prince, Haiti 2010	All affected	Study the characteristics, experiences and application of medical relief operations in tropical regions
Savage 2015 [37]	Peer-reviewed	Operational review	Typhoon (Haiyan) in Panay Island, Haiti 2013	All affected	Describe the experiences of the CAF medical response
Taylor 2007 [38]	Peer-reviewed	Operational review	Hurricane (Wilma) in Florida, USA 2005	Primarily elderly	Describe the outcomes of a MHU response

*Abbreviations: BDRCS* Bangladesh Red Crescent Societies, *CAF* Canadian Armed Forces, *DREF* Disaster Emergency Relief Fund, *DRT* Disaster Relief Team, *EMT* Emergency Medical Team, *ICRC* International Committee of the Red Cross, *IFRC* International Federation of Red Cross and Red Crescent Movement Societies, *NDMA* National Disaster Management Authorities, *PDMA* Provincial Disaster Management Authorities, *PRCS* Pakistan Red Crescent Society, *SAF* Singapore Armed Forces, *UNGRD* National Disaster Risk Management Unit

^*^Defined as minority groups and low-income populations [34]

### Themes

The literature highlighted seven themes: key characteristics, operational availability, services, benchmark indicator, staff, self-sufficiency and pre-deployment preparations. The information focus in each theme corresponded to WHO terminology why additional labels were not applied. The theme “benchmark indicator” refers to the patient count cared for by the mobile health units [10]. Data reflecting the mode of health service delivery and transportation were categorized as “key characteristics” [10]. Key characteristics and services were the most described themes, whereas operational availability, self-sufficiency and pre-deployment preparations were less documented. The quality of care was not commented in any document. The reporting frequencies of the themes are listed in Table 3.

**Table 3:** Reporting frequency of themes

Themes	Studies (*n* = 13)	Empirical sources
Key characteristics	13 (100%)	*Ahmed *et al*. (2008), Bouland *et al*. (2019), Broach *et al*. (2010), Ho *et al*. (2016), IFRC Bangladesh (2014), IFRC Colombia (2017), IFRC Pakistan (2017), Kim *et al*. (2010), Krol *et al*. (2007), Lateef *et al*. (2009), Li *et al*. (2012), Savage *et al*. (2015), Taylor *et al*. (2007)*
Services	13 (100%)	*Ahmad *et al*. (2008), Bouland *et al*. (2019), Broach *et al*. (2010), Ho *et al*. (2016), IFRC Bangladesh (2014), IFRC Colombia (2017), IFRC Pakistan (2017), Kim *et al*. (2010), Krol *et al*. (2007), Lateef *et al*. (2009), Li *et al*. (2012), Savage *et al*. (2015), Taylor *et al*. (2007)*
Benchmark indicator	12 (92%)	*Ahmed *et al. *(2008), Bouland *et al*. (2019), Broach *et al*. (2010), IFRC Bangladesh (2014), IFRC Colombia (2017), IFRC Pakistan (2017), Kim *et al*. (2010), Krol *et al*. (2007), Lateef *et al*. (2009), Li *et al*. (2012), Savage *et al*. (2015), Taylor *et al*. (2007)*
Staff	10 (77%)	*Ahmed *et al*. (2008), Broach *et al*. (2010), Ho *et al*. (2016), IFRC Bangladesh (2014), IFRC Colombia (2017), IFRC Pakistan (2017), Kim *et al*. (2010), Li *et al*. (2012), Savage *et al*. (2015), Taylor *et al*. (2007),*
Self sufficiency	6 (46%)	*Ahmed *et al*. (2008), Ho *et al*. (2016), IFRC Bangladesh (2014), IFRC Pakistan (2017), Krol *et al*. (2007), Lateef *et al*. (2009)*
Operational availability	3 (23%)	*Ahmed *et al*. (2008), Savage *et al*. (2015), Taylor *et al*. (2007)*
Pre-deployment preparations	3 (23%)	*Bouland *et al*. (2019), IFRC Pakistan (2017), Lateef *et al*. (2009)*

All 13 documents emphasized that the main objective for providing healthcare with mobile health units following natural disasters was to reach populations with limited access to fixed health facilities [26–38]. Target locations were described as “remote areas”, “dispersed clinical sites”, “isolated villages”, “remote mountain areas”, and “hard to reach outreach locations” [26, 27, 29, 34, 37]. Patients requiring higher levels of care and follow-up were transferred to functioning local health facilities [26–28, 32, 35, 36, 39, 40] and emergency care centres [38].

### Extracted data

#### Key characteristics

The MHUs were reported to arrive in affected locations between the 3rd and 35th day after the onset of the disasters and were operational for 2 days to 3 months [26–38]. An overview of the reported timeliness, duration of deployment and number of target locations are displayed in Table 4.

**Table 4:** Key characteristics

Document	Number of MHUs	Time of arrival*	Deployment duration (days)	Number of locations
Ahmad [26]	1	15	5	5
Bouland [27]	1	5	11	9
Broach [28]	1	15	4	4
Ho [29]	1	3	9	-
IFRC Bangladesh [30]	5	9	7	55
IFRC Colombia [31]	30	6	91	22
IFRC Pakistan [32]	3	24	99	71
Kim [33]	1	35	6	5
Krol [34]	2	7	16	23
Lateef [35]	1	-	9	10
Li [36]	1	-	15, 17, 26	-
Savage [37]	1–4	8	31	67
Taylor [38]	9	5	12	51

^*^Days after the onset of the disaster

The units relocated between target locations by road, air and water [26, 28–32, 34–38]. Multiple modes of transportation were used during the same deployment [26]. Jeeps, recreational vehicles and medical vans were used for relocation by road [29, 34, 38] and helicopters were used for transportation by air [27]. Characteristics of vehicles used for transportation on water were not specified. Two documents [29, 37] reported that predefined target locations were assigned by local health authorities, while one review [27] described MHUs relocating independently within assigned districts. Lateef et al. [35] reported that 1- 2 h were spent on traveling each day [35]. There were recurring descriptions of MHUs covering multiple locations daily [26, 30, 32, 35]. Ahmad et al. [26] described that medical services were provided for approximately 1,5–3 h at 2–3 locations each day, mainly during daylight hours. However, there was scarce information about the daily numbers of relocations and the time spent in each location. Transportation and a fast working pace were reported to contribute to staff fatigue [29, 32, 35]. Shift systems were implemented to mitigate exhaustion [29].

Mobile health units were described to operate within existing local structures, medically equipped vehicles and with in-house visits [26–38]. Flood relief centres, temples, community centres, warehouses, schools, retirement facilities and motels are examples of preexisting fixed facilities reported in the documents [26–28, 30, 32, 34, 36]. The medically equipped vehicles consisted of a registration area, nursing station and an examination room [34]. Broach et al. [28] reported that MHUs operated in spontaneously sprung tent camps following the 2010 Haiti earthquake, where there was large population displacement. Furthermore, MHUs provided support to fixed hospitals in need of assistance [27, 35]. Alternating between free-standing mobile treatment areas and functioning fixed facilities was highlighted to allow significant operational flexibility [27].

#### Operational availability

MHUs were operational on the same day or within one day of arrival according to two documents [26, 38]. Local health authorities informed local communities of the target locations and timeframe of MHU operations in advance [37]. However, most documents did not report on this theme.

#### Services

All 13 documents reported that primary healthcare (PHC), mental health consultations, vaccination and dispensing of medications were the most recurring services provided by MHUs [26–38]. Trauma presentations, such as lacerations, abrasions and fracture dislocations, constituted a minority of cases, especially for teams arriving weeks after the onset of the disasters [26, 28, 32, 33]. Primary healthcare services (PHC) mainly comprised health maintenance assessments, including blood pressure and blood glucose monitoring and medical screening examinations for communicable diseases (CDs) and noncommunicable diseases (NCDs). Upper respiratory tract infections and diarrheal diseases were common regardless of disaster type and setting [27, 28, 30–32, 34, 37, 38]. Ante-natal and post-natal check-ups were also conducted in MHUs [26, 28, 30, 32, 37, 38]. Deployment reports from flood disasters stressed the importance of considering the possible impact of weather-and climate-related factors and demographic characteristics on the health burden [30, 32]. Anxiety, acute stress syndrome and post-traumatic stress disorder were common presentations why mental health services, psychosocial support and supportive reassurance were provided [27, 30, 31, 34, 38]. In addition to antibiotics, prescription refills for chronic diseases were regularly dispensed [27, 32, 34, 36, 38]. Furthermore, health promotion activities, such as distribution of hygiene information posters and health education sessions, were conducted by assisting volunteers [26, 30, 32, 38].

#### Benchmark indicator

Benchmark indicators were mainly listed as full counts of consultations over a full deployment length, with an estimated patient count of 175 patients/day [25, 26, 30, 31, 34, 35]. Approximate daily estimates were reported in four documents, varying between 60 and 545 patients/day [28, 33, 37, 39]. However, deployment durations and number of personnel varied significantly between the teams [26, 27, 30–38]. It was not specified if the same patients had been consulted more than once by the same MHU.

#### Staff

The documents outline a distinct variation in medical competences and size of the healthcare teams. With a team comprising of 5 members, IFRC Colombia [31] had the smallest team in comparison to Li et al. [36], reporting 40–75 team members in CISAR operations. Medical staff consisted of doctors, nurses and pharmacists [26, 28–33, 36–38]. Team doctors were family physicians, emergency doctors, surgeons, orthopaedics and specialists in infectious diseases, dermatology and ear-nose-throat [26, 28–33, 36–38]. MHU operations were sometimes accompanied by interpreters, social workers, technicians, search- and rescue team members and representatives from local health authorities [30–32, 36]. Additionally, two documents described the implementation of designated team leaders [28, 38]. Team leaders were primarily nurse practitioners fluent in local language [28, 38]. Inclusion of female staff was described to contribute to gaining local trust [30, 32, 34]. Translators, security personnel, local drivers and locally connected social workers were highlighted to facilitate healthcare provision with MHUs [28, 29, 38].

#### Self-sufficiency

There were descriptions of regular replenishment of essential medicines and medical equipment by local health authorities [29, 32, 38]. The IFRC had MHUs fully equipped with essential medical supplies for 24 days of operations during floods in Colombia in 2017 [32]. There were no reports of MHUs self-sufficient over a full length of deployment.

#### Pre-deployment preparations

There was scarce information about pre-deployment preparations in the literature. Good relations with local health authorities and other assisting organizations prior to deployment were suggested to facilitate the entry of MHUs in disaster-affected communities [27, 32, 35]. Psychological counselling and vaccinations of staff were mentioned as essential pre-deployment preparations [35].

### Advantages and challenges of being mobile

The literature provided experience-based reflections on the operational aspects of mobile health service delivery in natural disasters, describing both advantages and limitations with the modality. Mobile health units were outlined as assets for healthcare provision to hard-to-reach populations with limited access to fixed healthcare facilities [26, 34, 37]. Early response with this modality was emphasized to help “reduce the burden of the local health authority” [26] and prevent possible development of illnesses and injuries into severe conditions in the absence of regular healthcare [38]. However, damaged roads, vehicle malfunctions and limited access to vehicles caused challenges in transporting medical supplies and reduced the flexibility of operations [30, 32, 34]. Additionally, limited local healthcare capacity, frequent relocations and disrupted infrastructure reduced referral and follow-up capacities [27, 32, 37]. Limitations mainly related to “communication, coordination and flexibility of operations” following hurricane Dorian in 2019 [27]. Communication problems were primarily attributed to poor network access in out-reach locations [32]. Moreover, the IFRC highlighted that the use of MHUs was costly due to high transportation costs following floods in Bangladesh in 2012 [30]. Small units with few medical personnel prevented MHUs from providing healthcare to large populations [37]. Coordination challenges between domestic medical coordination cells and the EMT Coordination Cell (EMTCC) was reported to result in conflicting tasking [27]. Team Rubicon, an EMT type 1 mobile, was tasked to assist at sites that were already covered by fixed facilities established by other organizations [27].

## Discussion

### Data limitations and need for systematic reporting

While the scoping review methodology allows broad inclusion of sources, literature addressing the use of MHUs in natural disasters was limited. With three operational reports eligible for inclusion, peer-reviewed publications comprised most of the literature scope. Additionally, the grey literature was published by the same organization despite databases of several organizations being queried [30–32]. Among the reviewed literature, one report described experiences of a classified EMT type 1 mobile [27]. The WHO EMT Initiative was asked for reports from prior deployments with EMT Type 1 mobile but were unable to provide any documents within the time frame of this study. The scarcity of publications by organizations engaged in disaster response highlights the discussion of transparency and accountability in emergency relief efforts [12]. With intentions to mitigate the health effects of disasters, there are many stakeholders engaged in healthcare assistance following natural disasters [41]. Engagement in disaster response is often branded with an urge to make a difference with altruistic motivation [7, 10, 42]. However, assistance that is not needs-based limit healthcare services from appropriately filling health care gaps [10, 12]. Optimal allocation and use of resources are imperative in resource-scarce settings [10]. Deployment reports provide valuable information about the capacities and limitations of MHUs in disasters. Thus, continuous evaluations are imperative to optimize the usefulness of MHUs in addressing health needs and guide the EMT Initiative in future deployments [20]. Lack of published deployment reports limits the chances of optimally assessing the usefulness of mobile health units in natural disasters why efforts must be made to make data available.

### MHU practices and adherence to WHO Minimum Standards

The MHUs were reported to arrive between the 3rd day and 5th week after the onset of the disasters [29, 33]. The findings align with prior research highlighting that international healthcare assistance does not arrive early enough to encounter trauma presentations following natural disasters [4, 6, 13]. According to the WHO minimum standards, EMT type 1 mobile should have the capacity to provide PHC and outpatient emergency care [10]. Regardless of the time of arrival and disaster type, reported health needs mainly related to PHC, including health maintenance assessments and medication dispensing for CDs and NCDs [26–38]. Our findings suggest that services of MHUs may primarily need to be oriented towards substituting inaccessible, regular health care rather than trauma care in natural disasters [26–38]. Reports about patient counts varied between 60 and 545 patients/day, meeting the WHO recommendation of at least 50 patients/day [10]. Information on self-sufficiency (46%), operational availability (23%) and pre-deployment preparations (23%) was limited and lacked necessary details to contextualize the data. Thus, it was not possible to assess their adherence to EMT minimum standards. It remains uncertain whether teams operated contrary to these standards or chose not to report.

Prior research and existing guidelines highlight that mobile healthcare is a flexible mode for health service delivery to remote areas [7, 10, 15, 43]. Accordingly, the findings of this study imply that mobile healthcare can be a good option to reach dispersed populations with limited access to fixed healthcare facilities [26–33, 35–38]. However, challenges related to transportation, coordination, communication, coverage, data collection and cultural barriers were reported, reinforcing the critiques of MHUs [7, 16, 17, 27, 30, 32, 34]. Reduced follow-up capacities due to disruption of local healthcare systems underscores the importance of comprehensive coordination of referrals. Limited mobility due to damaged roads and lack of appropriate vehicles could pose health care provision with MHUs vulnerable to aftershocks. Use of multiple modes of transportation and operational facilities may improve their capacity to adapt to unforeseen hazards and, as a result, contribute to strengthened surge capacity within the healthcare system in natural disasters.

### Optimizing MHU deployment and EMT guidelines: lessons from the literature

In advancing the usefulness of MHUs in natural disasters, several key improvement suggestions may be considered. The literature highlights a need for better coordination between international, national and ground-level coordination cells [27, 28]. To optimize resource allocation and avoid redundancy in health service provision, collaborative coordination between stakeholders may be useful. Additionally, strengthened regional and national mobile health capacities should be a central element in optimizing disaster preparedness and response. Comprehensive analysis of the main health needs, local healthcare capacity, and demographic and socioeconomic contexts is needed prior to deployment to assess whether MHUs are suited to address the health needs [26–30, 32, 34–37]. Good local relations and cultural sensitivity may facilitate healthcare provision through MHUs [28–30, 32, 34, 38]. Furthermore, MHUs must be adaptable to climate conditions, disaster characteristics and impact of potential aftershocks [26–33, 35–38]. Additionally, beyond mainly focusing on PHC, the literature underscores the importance of mental health services and psychosocial support in the scope of mobile health services [26–38]. Transportation challenges due to damaged infrastructure may necessitate a lightweight design with only essential equipment [26–29, 32]. To overcome challenging terrain and withstand aftershocks, back-up options for transportation can be useful. This approach allows flexibility in using multiple modes of transportation [26]. Exhaustion due to multiple daily relocations and high workload may reduce the quality of care and impair data collection. Staff fatigue can be mitigated with strategic planning and shift systems [10, 29, 32, 35]. Additionally, we encourage the use of standardized and user-friendly reporting protocols that reflect indicators outlined in guidelines and quality standards. This allows for experience based critical assessment and continuous evaluation of MHU operations.

### Methodological considerations and limitations

Thirteen documents were included in this review, of which grey literature comprised three operational reports produced by the IFRC. The limited representation of organizations may pose a risk of information bias, highlighting a need for enhanced transparency and possibly even independent evaluation of health efforts in disasters. Moreover, the small scope of thirteen documents and the exclusion of documents from less common disaster types, limit the possibility to draw general conclusions regarding the mobile mode of health service delivery in disasters. However, the findings provide valuable insight into documentation and reporting standards from prior deployments in natural disasters.

## Conclusions

Data on the use of mobile health units in natural disasters is scarce, with inconsistent reporting of key aspects. The reporting standards did not adhere to existing data collection protocols, stressing a need for uniform reporting. Mobile health units were described to improve healthcare access in hard-to-reach areas with dispersed populations. However, in the absence of functioning fixed healthcare facilities, they were reported to mainly address the normal burden of disease rather than emergency care. The literature highlights transportation, coordination, referral system and data collection as the main areas of improvement.

## Data Availability

No datasets were generated or analysed during the current study.

## Appendix A Search terms

**Table:**

Group A	"mobile health unit*" OR "mobile healthcare unit*" OR "mobile medical*" OR "mobile medical unit*" OR "mobile unit*" OR "ambulatory health unit*" OR "mobile clinic*" OR "mobile health clinic*" OR "mobile health van*" OR "portable health zone*" OR "portable health facili*" OR "emergency medical team*" OR "foreign medical team*" OR "mobile medical team*" OR "mobile health team*" OR "mobile care team*" OR "EMT type 1" OR "mobile hospital*" OR "field hospital*" OR "foreign field hospital*" OR "medical relief*"
Group B	"flood*" OR "storm*" OR "cyclone*" OR "hurricane*" OR "typhoon*" OR "earthquake*" OR "ground shaking*" OR "tsunami*" OR "extreme temperature*" OR "wave*" OR "extreme winter condition*" OR "landslide*" OR "rockslide*" OR "mudslide*" OR "drought*" OR "wildfire*" OR "fire*" OR "volcan*" OR "dry mas s movement*" OR "avalanche*" OR "rockfall*" OR "natural disaster*" OR "natural hazard*" OR “sudden onset disaster*"

Abbreviations: *EMT* Emergency Medical Team

## Abbreviations

ARHAP: African Religious Health Assets Programme
BDRC: Bangladesh Red Crescent Societies
CAF: Canadian Armed Forces
CD: Communicable disease
CDC: Centre for Disease Control
CINAHL: Cumulative Index to Nursing and Allied Health Literature
CISAR: China International Search and Rescue
CRASH: Centre de Réflexion sur l'Action et les Savoirs Humanitaires
CRED: Centre for Research on the Epidemiology of Disasters
DREF: Disaster Emergency Relief Fund
DRT: Disaster Relief Team
EM-DAT: Emergency Events Database
EMDS: Emergency Medical Team Minimum Data Set
EMT: Emergency Medical Team
EMTCC: Emergency Medical Team Coordination Cell
GHO: Global Health Observatory
ICRC: International Committee of the Red Cross
IFRC: International Federation of Red Cross and Red Crescent Movement Societies
JBI: Joanna Briggs Institute
MCH: Maternal and Child Health
MHU: Mobile Health Unit
MSF: Medicins sans Frontieres
NCD: Non communicable diseases
NDMA: National Disaster Management Authorities
PDMA: Provincial Disaster Management Authorities
PRCS: Pakistan Red Crescent Society
SAF: Singapore Armed Forces
TCA: Thematic Content Analysis
UNDRR: United Nations Office for Disaster Risk Reduction
UNGRD: National Disaster Risk Management Unit
UREPH: Unité de Recherche sur les Enjeux et les Pratiques Humanitaires
WHO: World Health Organization
